# The Importance of Estimates

**Published:** 1907-06-22

**Authors:** 


### Nursing Administration.

### THE IMPORTANCE OF ESTIMATES.

Among the 90 hospitals which furnished returns to "Burdett's Annual" relative to the cost of nursing last year, only 38, less than half, were in a position to estimate the annual cost of boarding the nurses. Among these are conspicuous the 11 principal London hospitals with medical schools, all of whom keep their accounts in such a way that the cost of boarding each section of the household can be readily disentangled. Deducting these the proportion of hospitals able to state the cost of feeding their nurses falls to one-third of those who furnished returns, and is probably less than a tenth of the total number.

It is curious that in many hospitals in which extreme care is taken to keep expenses down, the importance of arriving at a clear knowledge of the expenditure on the different departments of the household is not understood. Even really clever housekeepers to whom the question of what it costs to board the different sections of the institution is a matter of deep interest, recoil from the labour which they fancy it must entail; and the others, who are not perhaps very clever, but are often very painstaking, are unable to see the advantage of so ordering matters as to bring this knowledge within their grasp. Housekeepers, then, are divided into three classes: those who know, those who want to know, and those who neither know nor care to know.

It is very usual to evade the issue by preparing statements of the cost of board per bed or per head. Both of these are futile, but the cost of provisions per bed is not merely useless, but absolutely misleading, for any but the widest purposes of comparison. The amount of provisions consumed in the hospital may easily be divided by the number of occupied beds, and a rough average is thus obtained. But unless it be clearly specified how many persons other than patients are fed in the institution, nothing but confusion can result. The second plan —one which is often adopted in provincial hospitals —is to lump all the inhabitants of the institution together, patients, staff, nurses, and servants, and divide the cost of all provisions among them. When this reveals a low figure, it is assumed that all is going well, and the comparative amount spent on the different sections of the household is considered a matter of indifference. Yet not even the cleverest manager could tell how much it ought to cost to feed 200 people, some of whom are in strong health and working hard, while others are unable to take any solid food.

The variations in the cost of boarding different sections of the household may be ascertained by examining the figures of St. Thomas's Hospital. For the year 1902 the total cost of provisions was £11,360, and the daily average of persons boarded amounted to 709, including 465 in-patients. Now this total gives an average of £15 4s. per head per annum, or something like 10d. a day. Yet this amount was not the cost of boarding any one section of the household. The patients cost 8½d. a day; the nurses cost 1s. 3¼d. a day; the servants cost 8½d. a day; and the officers' board, including wine, costs 3s. a day. No purpose whatever could have been served by making a statement to the effect that the average cost of board in the hospital was 10d. Again, at St. Mary's, where the book-keeping is equally good, the cost of provisions in 1902 was £7,723, which, with a total of 414 persons boarded in the hospital, including a daily average of 248 patients, works out at an all-round cost of about 1s. a day. But the actual figures of the cost are widely different. The patients cost 10½d., the nurses cost 1s. 5d., the servants cost 6d., and the officers 2s. 1d. per day. Estimates of cost are obviously impossible unless the constituent parts of the household, with the corresponding charges involved, are allowed full weight. Suppose, for example, a provincial hospital were to rest satisfied with showing an average cost per day of 10d., basing the estimate on the average presented by St. Thomas's, as a hospital where the commissariat has a deservedly high reputation for economy and excellence. Such conclusions would be in the highest degree misleading, unless there were an equally large proportion of officers and nurses. In some provincial hospitals where the number of officers boarded is inconsiderable, the cost of board divided among all the inmates does not exceed 9d. a day, but to rest satisfied with this is to remain in ignorance of the true condition of affairs. It is possible to err on the side of parsimony as well as on the side of economy. The patients may be receiving inferior provisions, short allowance of milk, insufficient convalescent diet, while more than is required to make them comfortable is muddled away in the kitchen or among the staff. Or it may be that the patients' diet is needlessly embroidered with extras, while the nurses' table is pared of all but the coarsest fare. The matron, on whom ceaseless pressure is brought to bear to keep down expenses, is never sure of herself if she cannot produce the average cost of boarding each section of her household. Any little luxury which she can legitimately procure for her hard-working staff is apt to appear in the light of an extravagance unless she knows exactly what amount she has to depend on every week, and can plan her bill of fare to keep within her estimate. In hospitals of moderate size, where the number of medical officers is not large enough to constitute a separate section of the household, the division of the estimates into two distinct sections, for patients and for household, meets all requirements. But the circumstances under which the patients are fed, some receiving little else than milk, and even the convalescents being rigidly dieted, renders it quite impracticable that any accurate estimates of expenditure can be prepared unless the cost of their board be rigidly severed from that of the staff.

